# Estrogen Receptor (ER)-α36 Is Involved in Estrogen- and Tamoxifen-Induced Neuroprotective Effects in Ischemic Stroke Models

**DOI:** 10.1371/journal.pone.0140660

**Published:** 2015-10-20

**Authors:** Wei Zou, Chen Fang, Xiaofei Ji, Xiaofeng Liang, Yang Liu, Chao Han, Liang Huang, Qiqi Zhang, Hongyan Li, Yejun Zhang, Jinqiu Liu, Jing Liu

**Affiliations:** 1 Regenerative Medicine Centre, the First Affiliated Hospital of Dalian Medical University, Dalian 116011, China; 2 College of Life Science, Liaoning Normal University, Dalian 116081, China; 3 Liaoning Key Laboratories of Biotechnology and Molecular Drug Research and Development, Dalian, 116081, China; 4 Department of Cardiology, the First Affiliated Hospital of Dalian Medical University, Dalian 116011, China; National University of Singapore, SINGAPORE

## Abstract

The neuroprotection by estrogen (E2) and tamoxifen is well documented in experimental stroke models; however, the exact mechanism is unclear. A membrane-based estrogen receptor, ER-α36, has been identified. Postmenopausal-levels of E2 act through ER-α36 to induce osteoclast apoptosis due to a prolonged activation of the mitogen-activated protein kinase (MAPK)/extracellular signal-related kinase (ERK) signaling. We hypothesized that ER-α36 may play a role in the neuroprotective activities of estrogen and tamoxifen. Here, we studied ER-α36 expression in the brain, as well as its neuroprotective effects against oxygen and glucose deprivation (OGD) in PC12 cells. We found that ER-α36 was expressed in both rat and human brain. In addition, OGD-induced cell death was prevented by l nmol/L 17β-estradiol (E2β). E2β activates the MAPK/ERK signaling pathway in PC12 cells under basal and OGD conditions by interacting with ER-α36 and also induces ER-α36 expression. Low-dose of tamoxifen up-regulated ER-α36 expression and enhanced neuronal survival in an ovariectomized ischemic stroke model. Furthermore, low-dose of tamoxifen enhanced neuroprotective effects by modulating activates or suppress ER-α36. Our results thus demonstrated that ER-α36 is involved in neuroprotective activities mediated by both estrogen and tamoxifen.

## Introduction

The potential neuroprotective effects of estrogen (E2) have recently attracted much attention. Epidemiological evidence suggests that the morbidity and mortality of ischemic stroke are relatively low in premenopausal women compared with women who have experienced natural or surgical menopause [1, 2]. The neuroprotective effects of estrogen may be due to estrogen receptor (ER)-dependent genomic and non-genomic actions [3]. Nakamura et al. found that estrogen might protect the brain from damage caused by cerebral ischemia; this protective effect was mediated by the ER- signaling pathway [4]. In addition, the protective effects of estrogen on cortical tissue were decreased in mice treated with a selective ER-α antagonist, or following ER-α knockdown [5, 6]. However, Sampei et al. compared *ER-α66* gene-deficient mice with wild-type mice, and found no change in the degree of stroke injury between the two groups [7]. This indicated that ER-α66 might not mediate the neuroprotective effects of estrogen against stroke.

ER-α36, a novel variant of ER-α, was identified in ER-α66-negative breast cancer cells in 2005 [8]. ER-α36 is transcribed from a previously unidentified promoter located in the first intron of the *ER-α66* gene. It has been shown to colocalize and interact with caveolin-1 protein expressed in caveolae membranes, which may modulate a diverse range of signaling molecules [9]. ER-α36 has also been shown to mediate the effects of postmenopausal levels of estradiol (E2) on the proliferation, apoptosis, and differentiation of osteoblasts through transient activation of the mitogen-activated protein kinase (MAPK)/extracellular signal-related kinase (ERK) pathway [10]. Our recent report indicated that ER-α36 is expressed in the cortex and hippocampus of rats [11], which suggests that ER-α36 might also play an important role in nervous-system diseases.

Interestingly, tamoxifen (TAM), a selective estrogen receptor modulator, was found to modulate ER-α receptor-mediated cellular transcription and apoptosis. Low-dose TAM can significantly ameliorate neurological deficits and reduce the infarct size in a model of cerebral ischemia [12]; however, high-dose TAM may be associated with the increased risk of cerebrovascular disease [13]. Different patterns of co-regulator expression and post-translational ER modifications may be involved in the TAM-induced effects [14]. Furthermore, ER-α36 is believed to mediate the agonist activity of TAM by activating the MAPK/ERK signaling pathway [15]. These results suggest that ER-α36 might be involved in mechanisms that protect against cellular injury.

We hypothesized that ER-α36 mediated the protective effects of estrogen and TAM in stroke. Therefore, in this study we first explored ER-α36 expression in primary cultured cells and in the hippocampus. In addition, we investigated the mechanisms underlying estrogen signaling and revealed the involvement of the MAPK/ERK signaling pathway. Finally, we confirmed the expression of ER-α36 in human brain tissue and its colocalization with caveolin-1.

## Materials and Methods

Research on human subjects: This study was permitted and approved by the Dalian Medical University Ethics Committee. Written informed consent was obtained from every enrolled individual.

Animal research: This study was carried out in strict accordance with National Institutes of Health (NIH) recommendations set forth in the Guide for the Care and Use of Laboratory Animals. The Dalian Medical University Committee on the Ethics of Animal Experiments approved our protocol (permit number: L2012014). All surgery was performed after the administration of chloral hydrate anesthesia (400 mg/kg), and all efforts were made to minimize suffering. We examined the status of the animals every 30 min after surgery. After surgery, animals were housed individually in plastic cages with food and water, under a constant 12 h light/dark cycle. Amoxicillin was used to prevent wound infection. The mortality associated with MCAO surgery was about 30% before the 20-h time point. It was higher than a previous report (S1 File).

The Ethics Committees approved the experiments, including any relevant details regarding animal welfare, patient anonymity, drug side effects, and informed consent, as well as the guidelines and regulations according to which the experiments were performed.

### Animals

Three-month-old female Sprague-Dawley rats weighting 220–250 g (8–10 weeks) were housed under a 12:12 h light-dark cycle. The animals were fed a standard diet. Adult rats were anesthetized with chloral hydrate (400 mg/kg) and then killed by cervical dislocation. Newborn rats were decapitated without anesthesia.

### Human brain tissue

Brain tissue was provided by Professor Rongyao Liu (Department of Neurosurgery, the First Affiliated Hospital of Dalian Medical University). The brain tissue originated from 45–60 year-old patients with cerebral hemorrhage who had undergone neurosurgery. Their relatives gave written informed consent for the procedures, which were approved by the Ethics Committee on the Use of Human Subjects (Dalian Medical University and affiliated hospitals).

### Primary hippocampal neuron culture

Hippocampal neurons were harvested from the brains of newborn rats within 24 h of birth. Hippocampal tissue was placed in chilled D-Hanks’ Balanced Salt Solution (D-HBSS) and enzymatically dissociated using 0.125% trypsin for 10 min at 37°C. The cells were resuspended in Neurobasal®-A medium. Cells were seeded into poly-d-lysine (10 mmol/L)-coated 6-well culture plates at a density of 1 × 10^6^ cells/mL. Cultures were maintained in Neurobasal®-A medium and maintained at 37°C under an atmosphere of 5% CO_2_. The medium was changed 24 h after seeding; 50% of the medium was replaced every third day.

### Middle cerebral artery occlusion (MCAO) model

A monofilament was introduced into the internal carotid artery through an incision in the left common carotid artery. The middle cerebral artery was occluded for 4 h; symptoms were monitored every 30 min. No additional distress occurred apart from right-sided limb paralysis. The middle cerebral artery was reperfused for 20 h before the rats were sacrificed, and their status was monitored every 30 min.

### Nissl staining

Frozen sections were rinsed with phosphate-buffered saline (PBS) and then stained with 0.5% thionin. Sections were dehydrated using a graded series of alcohol solutions. After a rinse in dimethylbenzene, sections were sealed in neutral resin. The captured images were analyzed using Image-Pro Plus imaging software.

### PC12 cell culture

PC12 cells were purchased from the Shanghai Cell Corporation and cultured in Roswell Park Memorial Institute (RPMI) 1640 medium (Gibco) supplemented with 15% newborn calf serum (Gibco).

### Experimental design and drug treatment

Rats in the sham group were subjected to an abdominal incision. Six groups of ovariectomized (OVX) rats received the following surgery and drug treatments: (1) ovariectomy alone (*n* = 7); (2) ovariectomy and abdominal injection of vehicle (dimethyl sulfoxide, DMSO, 1 mL; *n* = 9); (3) ovariectomy and MCAO plus 1 mL DMSO injection (*n* = 9); (4) ovariectomy and MCAO plus 100 μg/kg TAM (*n* = 9); (5) ovariectomy and MCAO plus 1 mg/kg TAM (*n* = 9); and (6) ovariectomy and MCAO plus 5 mg/kg TAM (*n* = 9). TAM was dissolved in DMSO.

For MAPK activation assays, the cells were treated with vehicle (ethanol) and the indicated estradiol 17β (E2β) concentrations. To test the effects of different inhibitors, all inhibitors were added 1 h before E2β administration. After incubation in fresh medium overnight, cells were treated with 1 nmol/L E2β (Sigma-Aldrich) and 10 nmol/L IC162 at different time points prior to oxygen and glucose deprivation (OGD). In all cases, drug treatments were continued throughout the OGD period.

### OGD-treated cells

To achieve OGD, cells were cultured in a humidified anaerobic chamber with 5% CO_2_ and 95% N_2_. The culture medium was replaced with deoxygenated glucose-free minimum essential medium (MEM). An atmosphere of 0.3–0.5% oxygen at 37°C was maintained throughout the OGD period. After the OGD period, cultures were returned to normoxic conditions with RPMI 1640 medium.

### Immunofluorescence staining and confocal microscopy

Tissue sections were fixed with 4% parafomaldehyde, rinsed in PBS, and then blocked in PBS supplemented with 1% goat serum for 1 h at room temperature. Sections were subsequently incubated overnight with anti-ER-α36 antibody (1:300) and anti-NeuN antibody (1:100). After three 5-min washes in PBS, the sections were incubated for 1 h at room temperature in a solution of fluorescein isothiocyanate (FITC)-conjugated goat anti-rabbit immunoglobulin (IgG) antibody diluted 1:100 in PBS. After three washes in PBS, nuclei were stained for 5 min in Hoechst 33342 (1:1,000) and sections were evaluated.

Primary hippocampal neurons cultured on sterile glass cover slips were fixed in 4% paraformaldehyde in PBS for 24 h. After permeabilizing three times with 0.1% Triton X-100 at room temperature for 10 min, cells were washed in PBS for 5 min. They were then incubated in 3% H_2_O_2_ for 10 min, washed with PBS, blocked in 5% bovine serum albumin (BSA) for 1 h, and incubated overnight at 4°C with anti-ER-α36 antibody or anti-caveolin-1 antibody. After three washes in PBS, the cells were labeled with a FITC-conjugated secondary antibody and a Cy3-conjugated secondary antibody. Hoechst 33342 was used for nuclear staining. Microscopic analyses were performed using a fluorescence microscope.

### Cell viability assays

Cell viability was assessed using the 3-(4,5-dimethylthiazol-2-yl)-2,5-diphenyltetrazolium bromide (MTT) conversion method (Sigma, USA).

### Lactate dehydrogenase (LDH) leakage

LDH release was detected using a commercial kit purchased from Nanjing Jiancheng (Nanjing, China).

### DNA transfection and luciferase assays

For transient transfection assays, PC12 cells were seeded into 24-well plates and grown to 60–70% confluency in phenol-red-free medium plus 2.5% steroid-free FCS. Cells were washed and transiently transfected with 5 μg total plasmid with the FuGene® 6 reagent (Roche). We used a reporter plasmid containing two estrogen response elements (EREs; sequence from -331 to -289 of the chicken *vitellogenin A2* gene) placed upstream of the thymidine kinase promoter (2×ERE-tk-Luc; gift from Katarine Pettersson, Karolinska Institute, Stockholm). Expression vectors containing hER-α66 and hER-α36 were obtained from Professor ZhaoYi Wang. The luciferase assays were performed using a Luciferase Assay kit from Promega according to the manufacturer’s recommendations.

### Statistical analysis

Data were analyzed using Student’s *t*-tests, and are reported as mean ± standard deviation (*s*.*d*.). Mean values were obtained from a minimum of three independent experiments, and *p* < 0.05 was considered statistically significant.

## Results

**Fig 1:**
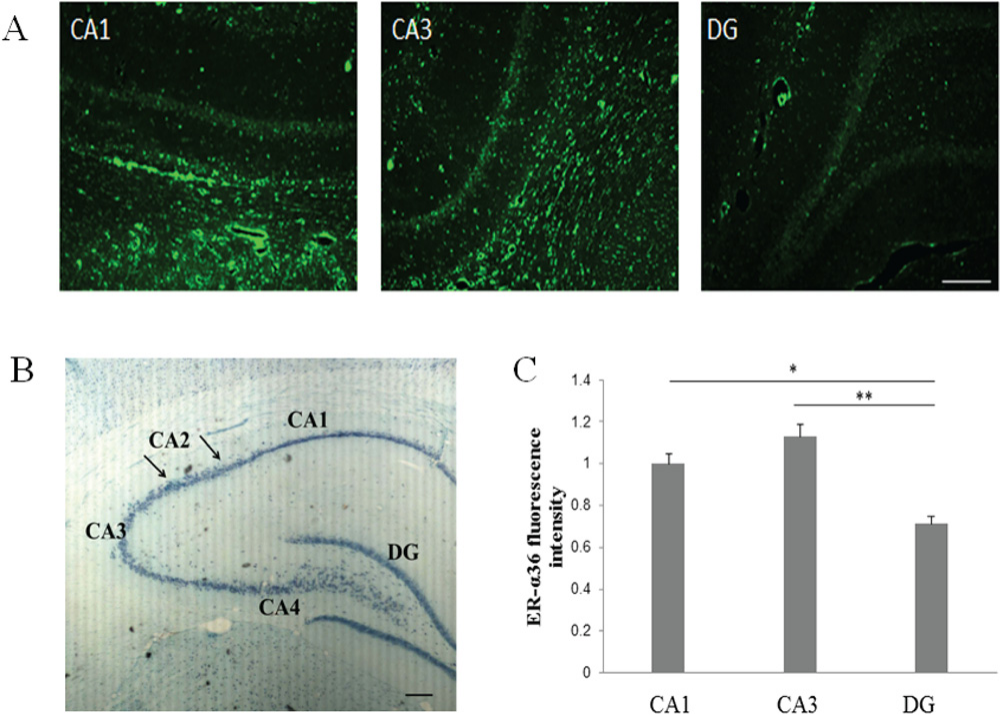
Estrogen receptor (ER)-α36 expression in the rat hippocampus. (**A**) Hyperfluorescence of ER-α36 expressed in the CA1 and CA3 regions of the rat hippocampus, and co-staining of ER-α36 with the neuronal marker, NeuN. (**B**) Nissl staining of the hippocampus. (**C**) Statistical analysis of ER-α36 expression. Scale bar = 200 μm (200×), *n* = 3. *p < 0.05, **p < 0.01.

1 The ER-α36 protein was highly expressed in pyramidal neurons.
  To study the hippocampal expression of ER-α36, we performed immunofluorescence to detect ER-α36 expression in the rat hippocampal CA1 and CA3 regions, and the dentate gyrus (DG; Fig 1). The results showed that ER-α36 was mainly located in hippocampal CA1 and CA3 pyramidal neurons. The fluorescence intensity of ER-α36 in the DG was lower than that observed in the CA1 and CA3 regions.

**Fig 2:**
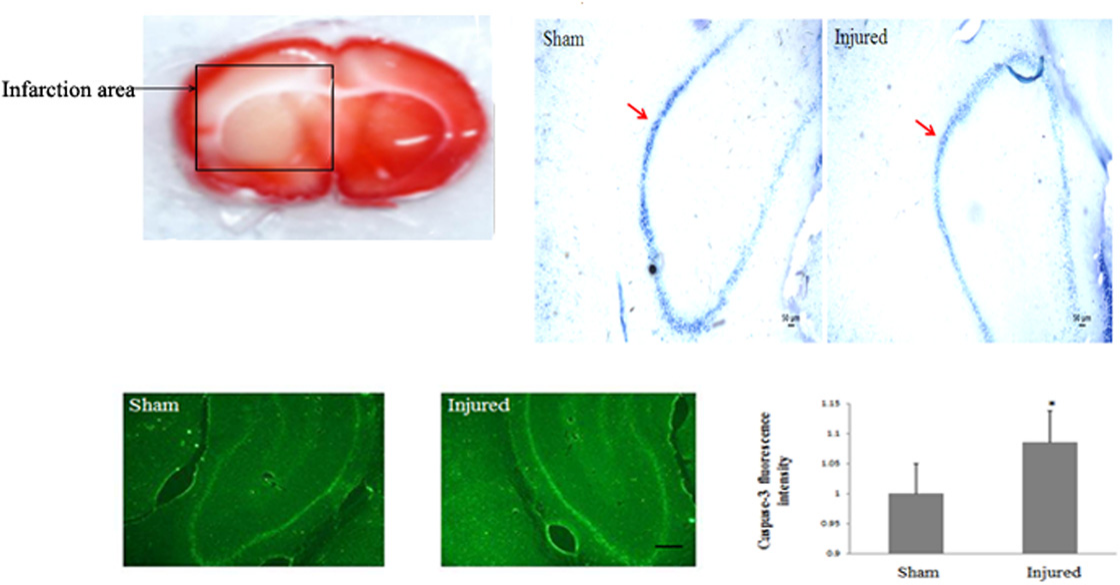
The ischemic brain of the MCAO model. Ischemia-reperfusion produced infarcts in the rat hippocampus, cortex and striatum. Red arrows show that ATP activity was decreased in the injured hippocampal CA1 region, as revealed by ATPase staining.

**Fig 3:**
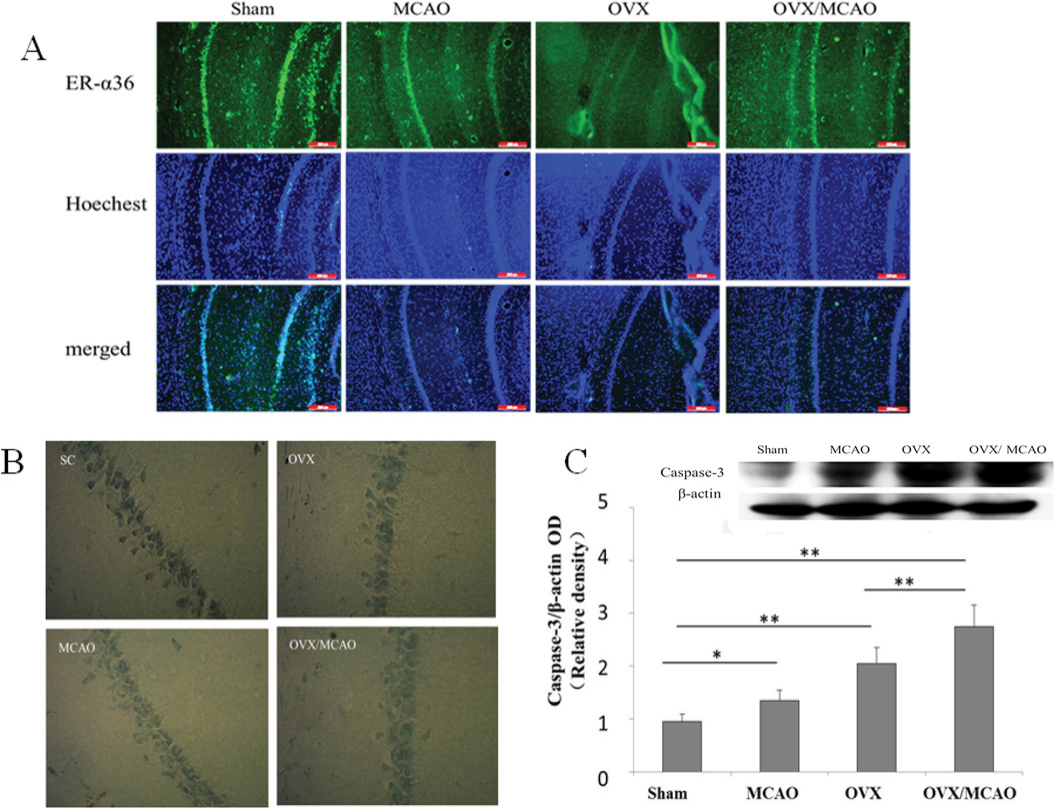
ER-α36 expression and cell injury in ischemic stroke models. (**A**) Immunofluorescence staining revealed that ER-α36 expression decreased in the hippocampal CA1 region of rats subjected to ovariectomy (OVX) and/or middle cerebral artery occlusion (MCAO). (**B**) The results of Nissl staining and (**C**) western blots of caspase-3 protein showed obvious neural injury in the OVX/MCAO group. Scale bar = 200 μm (200×), *n* = 3. *p < 0.05, **p < 0.01.

2 Decreased ER-α36 expression and significant neuronal injury occurred in the hippocampal CA1 region of OVX/MCAO rats.
  To study the effects of ischemia on ER-α36 expression, we used a fluorescence immunoassay to detect ER-α36 in normal female, OVX, and OVX/MCAO rats. The ischemic brain of the MCAO model is shown in Fig 2. ATPase staining revealed that ATP activity was decreased in the injured hippocampal CA1 region, which is a region sensitive to ischemic damage. As shown in Fig 3A, the ER-α36 fluorescence intensity was lower in MCAO rats compared with Sham rats. In OVX-treated rats, the fluorescence intensity was greater in MCAO rats compared with the OVX rats.

**Fig 4:**
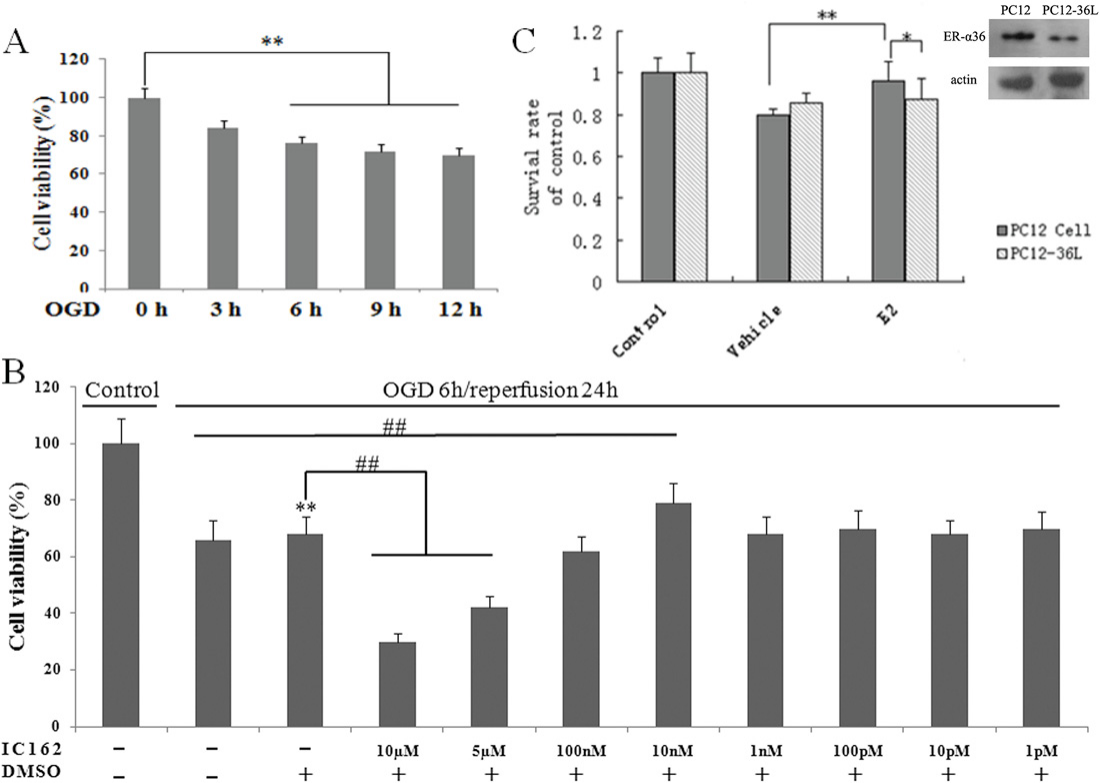
ER-α36 mediates the ability of estradiol 17β (E2β) to protect against oxygen and glucose deprivation (OGD). (**A**) OGD reduced PC12 cell viability after 6 h. (**B**) Different IC162 concentrations increased the protection against OGD in PC12 cells. (**C**) Decreased expression of ER-α36 after knockdown. ER-α36 knockdown promoted apoptosis in PC12 cells exposed to 6-h OGD, and weakened the neuroprotective effect of E2β. The results represent a percentage of the control values (no exposure to OGD), expressed as mean ± standard deviation (*s*.*d*.; *n* = 3). *p < 0.05, ** *p* < 0.01 vs. control; ^#^ *p* < 0.05, ^##^ *p* < 0.01 vs. vehicle-treated cells.

3 Nissl staining revealed that ischemia/reperfusion led to neuronal injury in the hippocampal CA1 region in MCAO rats; more severe injury was observed in OVX/MCAO rats (Fig 3B). Furthermore, western blotting results showed that caspase-3, an important indicator of apoptosis in ischemia/reperfusion injury, was present at a low level in the sham group, but was significantly increased in the OVX and OVX/MCAO groups (Fig 3C).
4 ER-α36 modulated E2β signaling to protect against OGD.
  The results showed that exposure of PC12 cells to 6 or 12 h OGD significantly decreased cell viability to approximately 20% and 30%, respectively (*t*-test, *p* = 0.007, Fig 4A). Cell death represented by LDH release into the medium showed similar results (a 76% increase). An ER-α36-selective agonist, IC162, was used to investigate whether ER-α36 was involved in the E2β-induced protection of PC12 cells [16]. PC12 cells treated with 10 nmol/L IC162 had a significantly higher survival rate than cells treated with vehicle (Fig 4B). ER-α36 was knocked down in PC12 cells to determine whether the ER-α36-mediated protection against OGD was antagonized by E2β. The results showed that the survival rate of ER-α36-knockdown cells (PC12-36L) was lower than that of wild-type PC12 cells. The MTT assay was used to determine the viability of wild-type and PC12-36L cells that had been pretreated with E2β and exposed to OGD for 6 h. In contrast to wild-type cells, E2β did not exert a protective effect on PC12-36L cells (Fig 4C).

**Fig 5:**
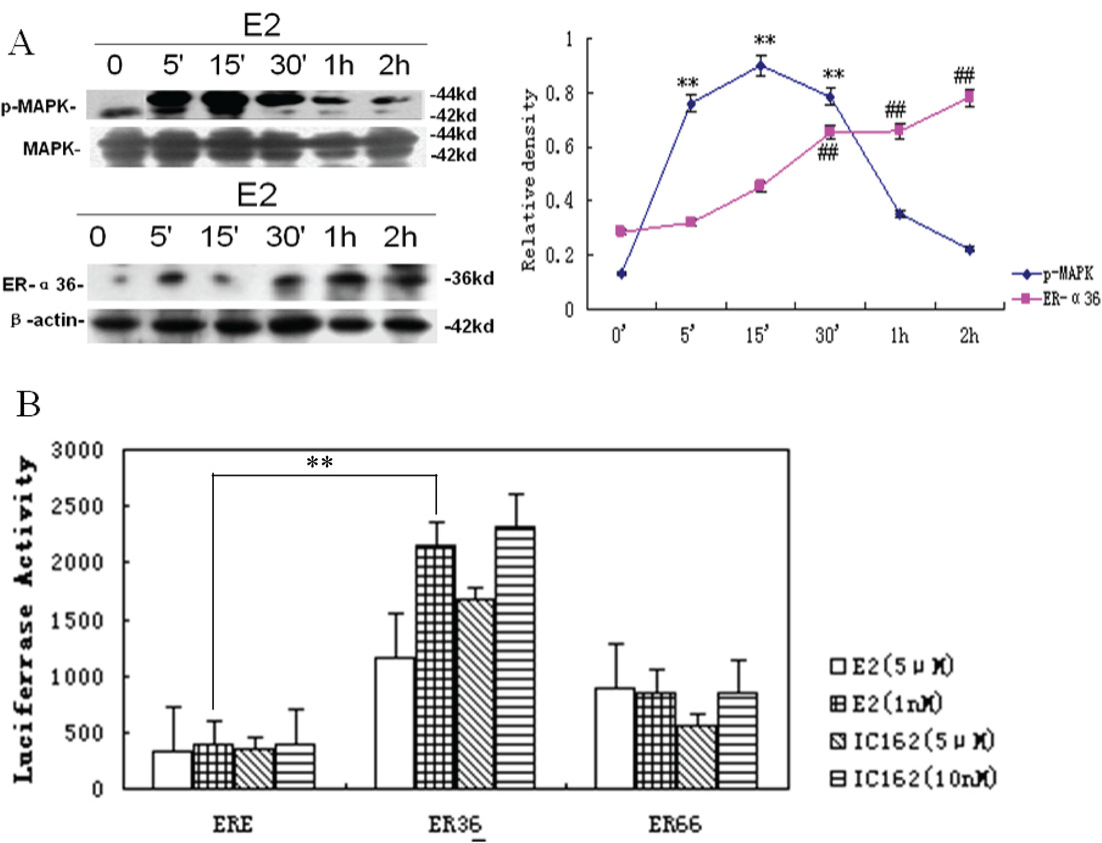
ER-α36 is involved in mitogen-activated protein kinase (MAPK) signaling pathway activation. (**A**) Low-dose E2β-induced activation of MAPK in PC12 cells under non-injury conditions. (B) Low-dose E2β induced activation of the ER-α36 promoter. The results represent a percentage of the control values (no exposure to OGD), expressed as mean ± standard deviation (*s*.*d*.; n = 3). *p < 0.05 vs. control.

5 MAPK/ERK was involved in estrogen-induced ER-α36 promoter activity.
  In the experiments described above, we observed that the treatment of cells with estrogen increased their resistance to OGD. We then examined estrogen-induced phosphorylation of MAPK/ERK, a non-genomic estrogen-signaling event. As shown in Fig 5A, we found that E2β induced the phosphorylation of stress-activated protein kinase (SAPK)/c-Jun N-terminal kinase (JNK), which activated the MAPK/ERK signaling pathway after 5 min. Activation of the MAPK/ERK signaling pathway reached its peak after 15 min and lasted for 1 h. In addition, we also found that 1 nmol/L E2β increased ER-α36 promoter activity; however, this did not occur at l5 μmol/L. IC162 at 1 nmol/L, but not at 5 μmol/L, also induced phosphorylation of the MAPK/ERK signaling pathway and activated the ER-α36 promoter (Fig 5B).

**Fig 6:**
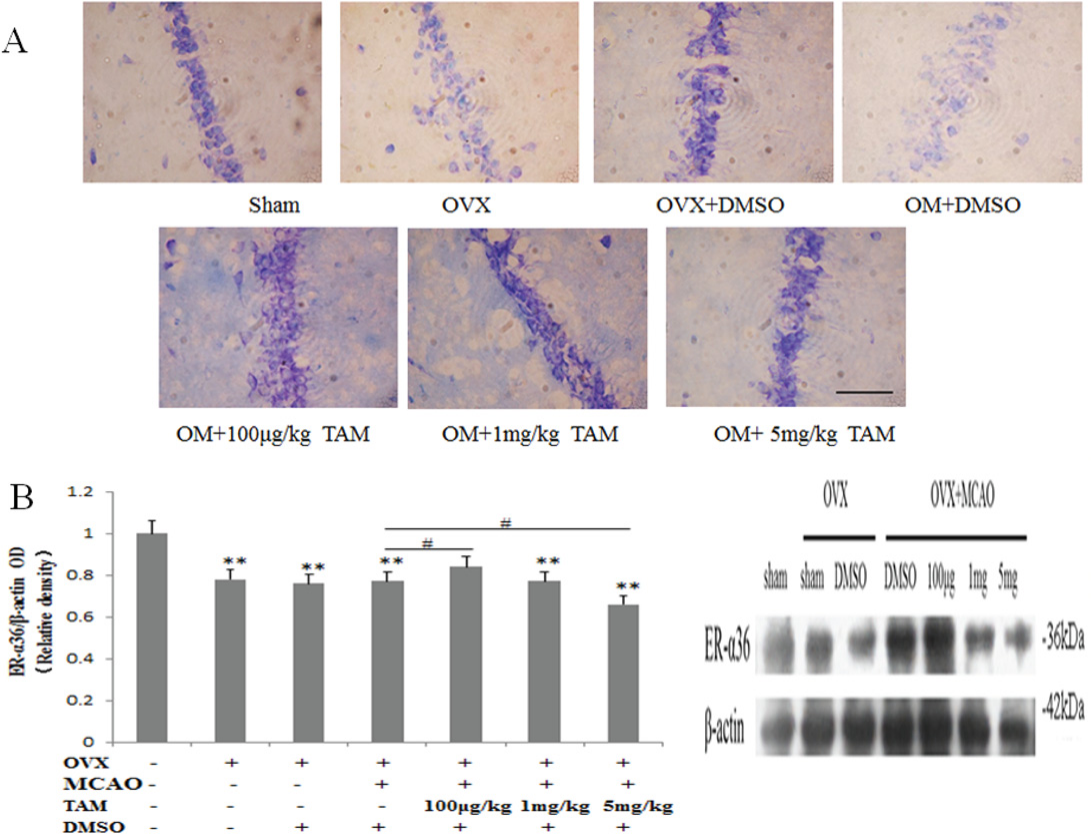
Tamoxifen (TAM) promotes ER-α36 expression to mediate E2β-induced neuroprotection. (**A**) The neuroprotective effect of TAM in hippocampal CA1 neurons following MCAO. OM stands for ovariectomy with middle cerebral artery occlusion. Scale bar = 50 μm (400×). (**B**) ER-α36 expression in rat hippocampal CA1 neurons after TAM treatment. Scale bar = 200 μm (200×). *p < 0.05, **p < 0.01 vs. sham group; #*p* < 0.05 vs. DMSO group.

6 ER-α36 expression in TAM-induced neuroprotective effects.
  To determine the effect of TAM on CA1 hippocampal neurons, we first visualized neurons using Nissl staining. After MCAO, the intensity of Nissl staining of CA1 neurons was decreased compared with the sham group. However, a 7-day course of TAM injections (100 μg/kg, 1 mg/kg, or 5 mg/kg) mitigated the loss of Nissl staining. The most effective TAM concentration was 1 mg/kg (Fig 6A). Western blotting results showed that hippocampal ER-α36 expression was down-regulated in ovariectomized rats. ER-α36 expression was higher after TAM treatment compared with the sham group. ER-α36 was maximally increased in the group that received a 100 μg/kg TAM injection, and the ER-α36 level decreased with increasing concentrations of TAM (Fig 6B). This study is the first to demonstrate that ER-α36 is upregulated after TAM treatment.

**Fig 7:**
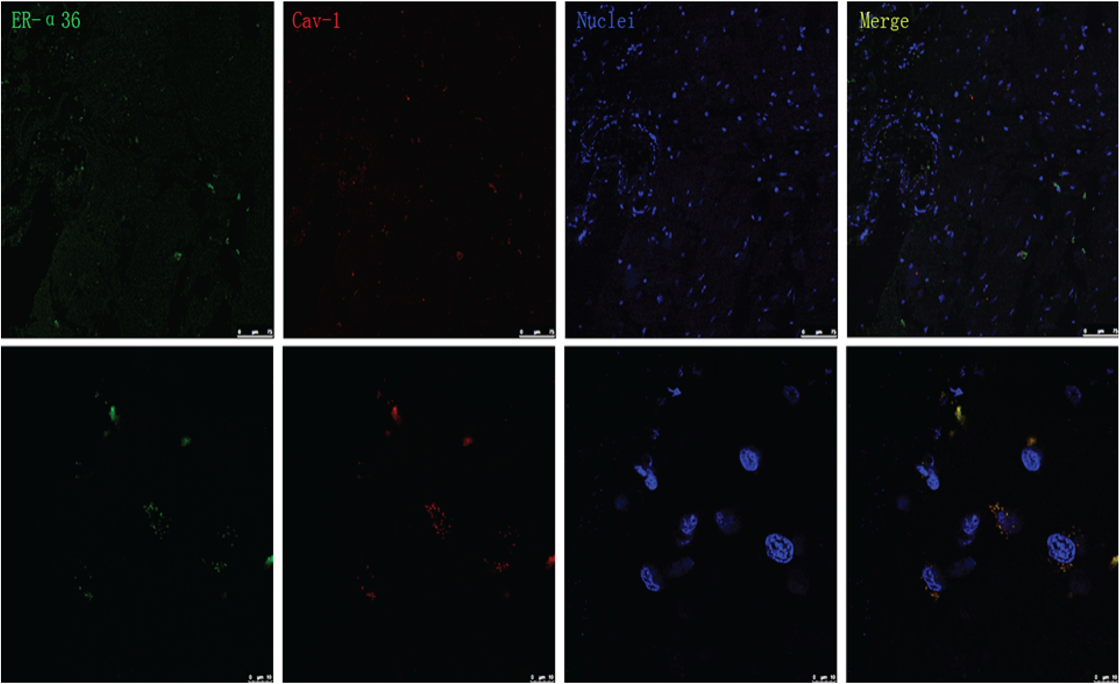
Expression of ER-α36 and caveolin-1 protein in the human cerebral cortex. Immunocytochemical staining of ER-α36 (green), caveolin-1 (red), and nuclei (blue) in brain slices at different magnifications. Scale bars = 75 μm/10 μm.

7 ER-α36 expression in the human cerebral cortex and its colocalization with caveolin-1.
  Immunofluorescence was performed to confirm the expression of ER-α36 in the human brain. We found that that ER-α36 was predominantly located in the cerebral cortex (Fig 7). In addition, ER-α36 was mainly expressed on the cell membrane and in the cytoplasm, and colocalized with the caveolin-1 protein.

## Discussion

Our results have shown for the first time that a new variant of ER-α, ER-α36, can not only be detected in different regions of the adult rat brain **(S1 Fig)**, including different regions of hippocampus, but also in human brain tissue. Previous studies had shown that ER-α is expressed in the hippocampus and cortex [10], but the distribution of ER-α36 in the central nervous system (CNS) was still unknown. We have demonstrated the presence of a novel plasma membrane-associated estrogen receptor (ER-α36) in the hippocampus, cortex, cerebellum, and diencephalon. ER-α36 was particularly highly expressed in the hippocampus, cortex, and cerebellum, suggesting that ER-α36 may be involved in CNS regulation. ER-α36 was widely expressed in the CNS, and therefore may be a target for estrogen therapy in stroke.

**Fig 8:**
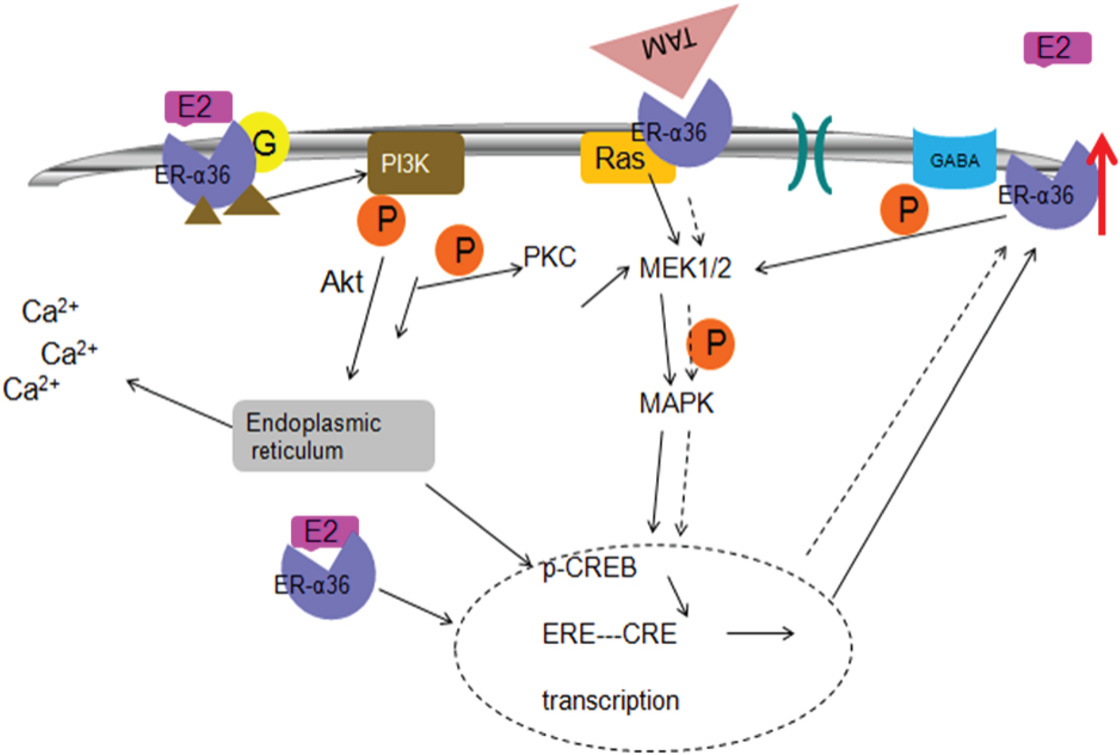
Diagram of the neuroprotective effects of estrogen and TAM.

In the present study, ER-α36 was shown to mediate the increased viability of PC12 cells that was induced by E2 during OGD. E2 was effective at a concentration of 1 nmol/L, which is lower than the circulating levels found in animals during the estrous cycle, and is consistent with concentrations of E2 that are known to bind with high affinity to its receptors. The data presented here confirm that E2β failed to protect ER(-α66)-negative PC12 cells at higher concentrations, which is also consistent with a previous report demonstrating that certain pharmacological concentrations of E2β inhibit the growth of ER-negative cells [17]. In addition, the fluorescence intensity was lower in MCAO rats compared with the Sham group. In OVX-treated rats, the fluorescence intensity was greater in MCAO rats compared with the OVX rats. These results indicated that ER-α36 might mediate the neural protection afforded by low concentrations of estrogen. The activation of MAPK/ERK signaling and ER-α36 expression mediated by non-genomic estrogen signaling exhibited a biphasic pattern, which provided a molecular explanation for the biphasic mitogenic estrogen signaling observed previously. Moreover, in ovariectomized rats with decreased estrogen levels, TAM could increase the expression of ER-α36 to enhance the neuroprotective effects of estrogen (Fig 8). This study may lead to a novel use of TAM for neuroprotection.

In previous studies, TAM was believed to modulate non-ER-α receptor-mediated neuroprotection via its antioxidant effects and free radical scavenging [18–20]. These studies used ICI 182,780, a kind of ‘pure’ anti-estrogen, as a ligand for ERs and found that TAM still exerted neuroprotective effects on the treated cells. After ER-α36 was isolated, Kang et al. found that ICI 182,780 failed to induce the degradation of this type of ER receptor [21]. However, ER-α36 can mediate the agonist activity of TAM by activating the MAPK/ERK signaling pathway in cells that do not express ER-α66 [15]. These results are consistent with our study. Therefore, in addition to its antioxidant effects, TAM may provide neuroprotection that is mediated by ER-α36.

A unique aspect of this study was its focus on PC12 cells, which lack ER-α66. It is known that mitogenic estrogen signaling does not contribute to the development or progression of ER-negative breast cancer that do not express ER-α66. However, early findings have demonstrated that ovariectomy could prevent the development of both ER-positive and ER-negative breast cancers [17]. ER-negative breast cancer cells that lack ER-α66 but express ER-α36 exhibit potent mitogenic estrogen signaling in vitro and in vivo [22]. PC12 cells were chosen for our study because they do not have ER-α66, but highly express ER-α36, which eliminates the possibility of ER-α66 involvement. In addition, ER-β reportedly has no effect on PC12 cells. The estrogen signaling response curve in these cells was biphasic, which led us to investigate the molecular mechanisms involved in biphasic estrogen signaling.

In conclusion, the present study has demonstrated that estrogen protects PC12 cells from OGD-induced damage via ER-α36-dependent mechanisms. Furthermore, low-dose TAM enhanced neuroprotection by regulating ER-α36 expression. These findings have clearly shown the direct protective effect of estrogen against ischemic injury in neuron-like cells in vitro and demonstrated a novel mechanism for E2-mediated neuroprotection. This sheds new light on possible therapeutic strategies for ischemic stroke.

## Supporting Information

S1 Fig Western blot results of ER-α36 expression in rat brain. (TIF)

S1 File The mortality rate of MCAO in other report was less than 30%. (PDF)
