# Supplementary figures and images for: Estrogen Receptor (ER)-α36 Is Involved in Estrogen- and Tamoxifen-Induced Neuroprotective Effects in Ischemic Stroke Models
Source: PLoS One. 2015 Oct 20;10(10):e0140660. doi: 10.1371/journal.pone.0140660 (PMC4618921; doi:10.1371/journal.pone.0140660)

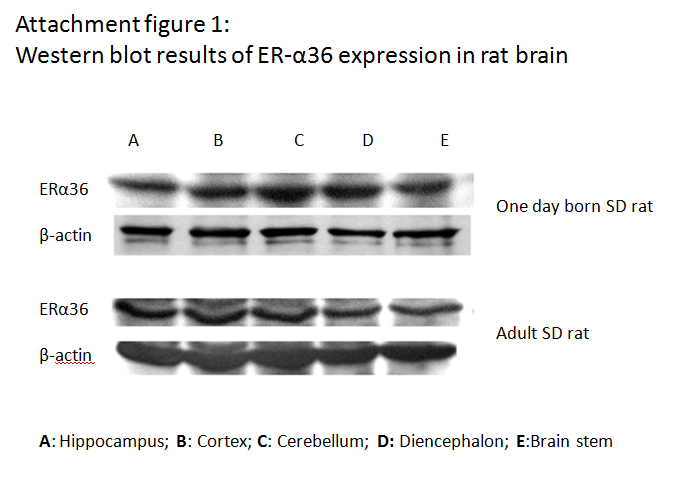

Supplement: S1 Fig — (TIF) [file pone.0140660.s001.tif]
