# Supplementary material for: Estrogen Receptor (ER)-α36 Is Involved in Estrogen- and Tamoxifen-Induced Neuroprotective Effects in Ischemic Stroke Models
Source: PLoS One. 2015 Oct 20;10(10):e0140660. doi: 10.1371/journal.pone.0140660 (PMC4618921; doi:10.1371/journal.pone.0140660)
Supplement: S1 File — (PDF) [file pone.0140660.s002.pdf]

Stroke
JOURNAL OF THE AMERICAN HEART ASSOCIATION

American Stroke Association
A Division of American Heart Association

Reversible middle cerebral artery occlusion without craniectomy in rats

EZ Longa, PR Weinstein, S Carlson and R Cummins

Stroke 1989, 20:84-91
doi: 10.1161/01.STR.20.1.84
Stroke is published by the American Heart Association. 7272 Greenville Avenue, Dallas, TX 72514
Copyright © 1989 American Heart Association. All rights reserved. Print ISSN: 0039-2499. Online ISSN: 1524-4628

The online version of this article, along with updated information and services, is located on the World Wide Web at:
http://stroke.ahajournals.org/content/20/1/84

Subscriptions: Information about subscribing to Stroke is online at
http://stroke.ahajournals.org//subscriptions/

Permissions: Permissions & Rights Desk, Lippincott Williams & Wilkins, a division of Wolters Kluwer Health, 351 West Camden Street, Baltimore, MD 21202-2436. Phone: 410-528-4050. Fax: 410-528-8550. E-mail:
journalpermissions@lww.com

Reprints: Information about reprints can be found online at
http://www.lww.com/reprints

Downloaded from http://stroke.ahajournals.org/ by guest on October 25, 2011

Reversible Middle Cerebral Artery Occlusion Without Craniectomy in Rats

Enrique Zea Longa, MD, Philip R. Weinstein, MD,
Sara Carlson, BS, and Robert Cummins, MS

To develop a simple, relatively noninvasive small-animal model of reversible regional cerebral ischemia, we tested various methods of inducing infarction in the territory of the right middle cerebral artery (MCA) by extracranial vascular occlusion in rats. In preliminary studies, 60 rats were anesthetized with ketamine and different combinations of vessels were occluded; blood pressure and arterial blood gases were monitored. Neurologic deficit, mortality rate, gross pathology, and in some instances, electroencephalogram and histochemical staining results were evaluated in all surviving rats. The principal procedure consisted of introducing a 4-0 nylon intraluminal suture into the cervical internal carotid artery (ICA) and advancing it intracranially to block blood flow into the MCA; collateral blood flow was reduced by interrupting all branches of the external carotid artery (ECA) and all extracranial branches of the ICA. In some groups of rats, bilateral vertebral or contralateral carotid artery occlusion was also performed. India ink perfusion studies in 20 rats documented blockage of MCA blood flow in 14 rats subjected to permanent occlusion and the restoration of blood flow to the MCA territory in six rats after withdrawal of the suture from the ICA. The best method of MCA occlusion was then selected for further confirmatory studies, including histologic examination, in five additional groups of rats anesthetized with halothane. Seven of eight rats that underwent permanent occlusion of the MCA had resolving moderately severe neurologic deficits (Grade 2 of 4) and unilateral infarcts averaging 37.6±5.5% of the coronal sectional area at 72 hours after the onset of occlusion. Five rats underwent the same procedure after bilateral vertebral artery occlusion was performed to reduce collateral blood flow. Only two of these five rats survived 72 hours; the neurologic deficits progressed from Grade 2.5 to 3, and the infarcts were larger than after MCA occlusion alone. In two groups of rats, the suture was withdrawn from the ICA to permit reperfusion after 2 or 4 hours of ischemia. Five of 10 rats subjected to 4-hour temporary MCA occlusion and one of six rats subjected to 2-hour temporary MCA occlusion did not survive 72 hours after the onset of occlusion. Infarct areas in surviving rats after 2-hour temporary MCA occlusion were 15.7% smaller than after permanent MCA occlusion, but the neurologic deficit was not significantly reduced by reperfusion. Fatal intracranial hemorrhage occurred in only two of 71 rats after occlusion of the MCA with an intraluminal suture. The results in the six sham-occluded rats showed that occlusion of the extracranial carotid branches, dissection of the cervical ICA, and placement of an intraluminal suture in the ECA did not produce stroke. This model provides a reliable method for studying reversible regional ischemia in rats without craniectomy. (*Stroke* 1989;20:84–91)

The pathophysiology of cerebral ischemia has been studied extensively in rats with various methods, including multiple vessel occlusion, hypotension, and hypovolemia, to produce global alteration in cerebral blood flow and metabolism.[1-8] The search for a reliable, less invasive rat stroke model of temporary regional ischemia has been prompted by the extensive neurochemical data already available in rats, the rising cost of experiments with larger animals, and the limitations of other rodent models of focal cerebral ischemia, such as occlusion of the common carotid artery (CCA)[9] or middle cerebral artery (MCA)[10] in gerbils. A direct microsurgical technique for permanently occluding the MCA through a craniectomy[11-13] and an indirect method of producing hemi-

From the Department of Neurological Surgery, School of Medicine, University of California, San Francisco; San Francisco Veteran's Administration Hospital; and the Microsurgery Laboratory, Ralph K. Davies Medical Center, San Francisco, California.

Supported by the Veterans Administration Research Service.

Address for correspondence: Philip R. Weinstein, MD, Department of Neurological Surgery, The Editorial Office, 1360 Ninth Avenue, Suite 210, San Francisco, CA 94122.

Received August 1, 1987; accepted July 7, 1988.

Downloaded from http://stroke.ahajournals.org/ by guest on October 25, 2011

spheric ischemia and reperfusion[14] in rats have also been described.

We sought to develop a model of reversible regional cerebral ischemia in rats without craniectomy based on advancing an intraluminal suture from the internal carotid artery (ICA) to occlude the origin of the MCA.[15] We also tested the effects of varying suture size, rat weight, and alternative methods of reducing collateral circulation to supplement the effect of MCA occlusion. We describe our preliminary experiments to select the optimal surgical procedure and present the neurologic, electroencephalographic (EEG), and pathologic findings we obtained in confirmatory studies with this preparation.

Materials and Methods

Adult male Sprague-Dawley rats weighing 400–500 g were anesthetized with 80–100 mg/kg i.p. ketamine hydrochloride and 5 mg/kg i.p. acepromazine maleate. A PE-50 catheter was introduced into the femoral artery for continuous monitoring of arterial blood pressure and sampling of blood for analysis of blood gases and hemoglobin concentration. The rats' body temperature was maintained at 37° C with an infrared heat lamp and a heating pad. In one group of rats, monopolar EEG recordings were obtained with transcutaneous needle electrodes placed 3 mm lateral to the sagittal suture in the parietal and frontal regions; a reference electrode was placed adjacent to the nasion. A Neurotrac EEG recorder and spectral analyzer (Interspec, Conshohocken, Pennsylvania) or a Grass Model 79 polygraph (Quincy, Massachusetts) with P511 amplifiers and an A.R. Vetter Co. C-4 tape recorder (Rebersburg, Pennsylvania) were used. The filter band pass settings were 0.3–300 Hz.

The vascular occlusive procedures performed in each of eight groups of rats in the preliminary studies are shown in Table 1. Groups 6, 7, and 8 were included to control for possible hemodynamically significant effects of occluding the vertebral artery or external carotid artery (ECA).

The basic surgical procedure consisted of blocking blood flow into the MCA with an intraluminal suture introduced through the extracranial ICA. Additional extracranial vessels, including the left ICA, right ECA, and both vertebral arteries, were occluded to reduce collateral blood flow to the MCA territory.

Under the operating microscope, the right CCA was exposed through a midline incision; a self-retaining retractor was positioned between the digastric and sternomastoid muscles, and the omohyoid muscle was divided. The occipital artery branches of the ECA were then isolated and coagulated (Figure 1, left). Next, the superior thyroid and ascending pharyngeal arteries were dissected and coagulated. The ECA was dissected further distally and coagulated along with the terminal lingual and maxillary artery branches, which were then divided. The ICA was isolated and carefully

Table 1. Experimental Groups, Reversible Middle Cerebral Artery Occlusion Without Craniectomy in Rats

Group	*n*	Procedure
Preliminary studies (N=60)		
1	8	R MCO
2	8	R MCO+L ICO
3	12	R MCO 24 hr after B VO
4	8	4-hr R MCO 24 hr after B VO
5	6	2-hr R MCO
6	6	B VO
7	6	ECO
8	6	ECO 24 hr after B VO
Confirmatory studies (N=34)		
A	5	Suture in ECO (sham operation)
B	8	Permanent R MCO
C	6	2-hr R MCO
D	10	4-hr R MCO
E	5	Permanent R MCO immediately after B VO

R MCO, right middle cerebral artery occlusion by intraluminal suture; L ICO, left internal carotid artery occlusion by intraluminal suture; B VO, bilateral vertebral artery occlusion by transection of terminal branches and placement of an intraluminal suture; ECO, external carotid artery occlusion by intraluminal suture. Group 5 was used for electroencephalographic studies only; Groups 6, 7, and 8 were control groups.

separated from the adjacent vagus nerve. Further dissection identified the ansa of the glossopharyngeal nerve at the origin of the pterygopalatine artery; this posteriorly directed extracranial branch of the ICA was ligated with 7-0 nylon suture close to its origin. At this point, the ICA is the only remaining extracranial branch of the CCA.

Next, a 6-0 silk suture was tied loosely around the mobilized ECA stump, and a curved microvascular clip was placed across both the CCA and the ICA adjacent to the ECA origin. A 5-cm length of 4-0 monofilament nylon suture, its tip rounded by heating near a flame, was introduced into the ECA lumen through a puncture or through one of the terminal branches of the ECA. (In some smaller rats in the preliminary studies, a 5-0 intraluminal suture was used when initial attempts to advance a 4-0 suture proved unsuccessful.) The silk suture around the ECA stump was tightened around the intraluminal nylon suture to prevent bleeding, and the microvascular clip was removed. The nylon suture was then gently advanced from the ECA to the ICA lumen; the position of the suture within the ICA lumen could be seen as it reached the base of the skull. After a variable length of nylon suture had been inserted into the ECA stump, resistance was felt and a slight curving of the suture or stretching of the ICA was observed, indicating that the blunted tip of the suture had passed the MCA origin and reached the proximal segment of the anterior cerebral artery (ACA), which has a smaller diameter (Figure 1, right). At this point, the intraluminal

Downloaded from http://stroke.ahajournals.org/ by guest on October 25, 2011

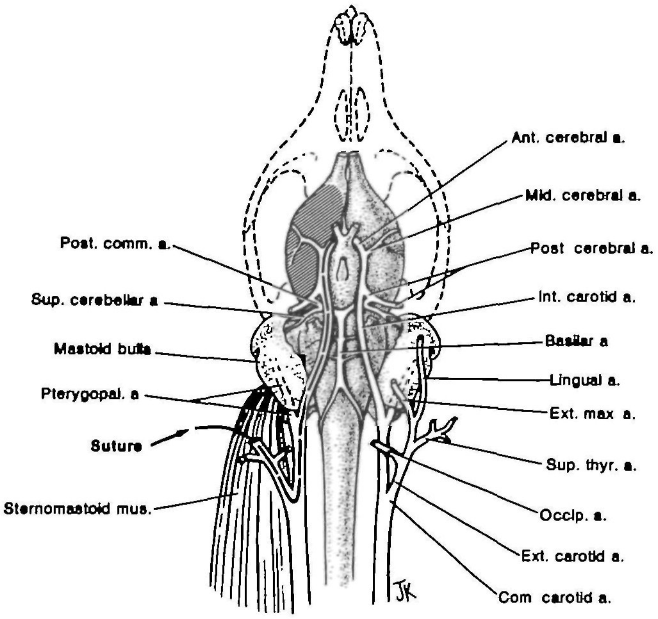

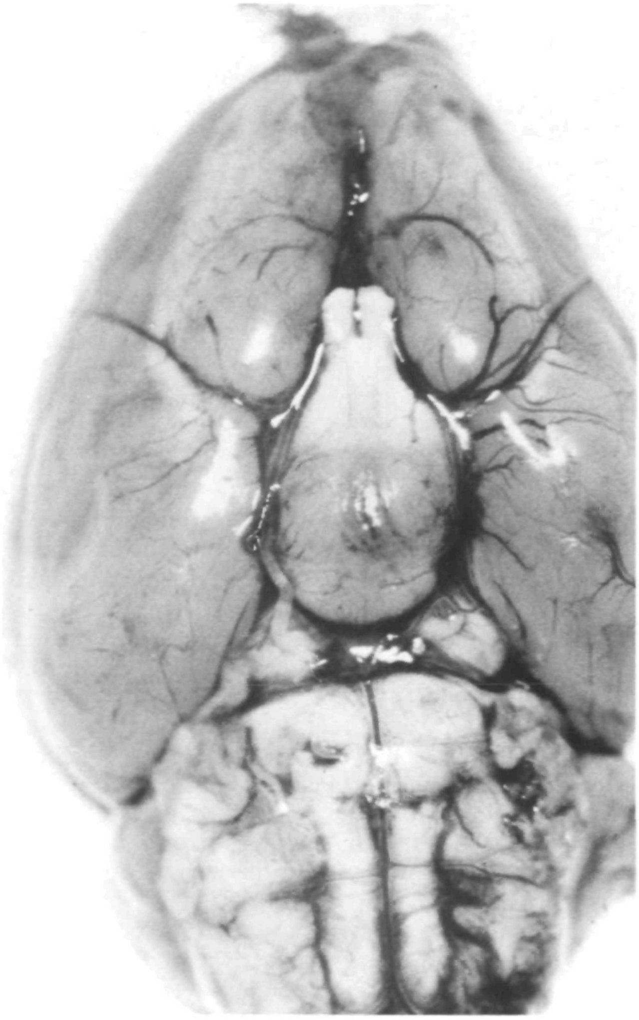

FIGURE 1. Left: *Diagram of cerebrovascular anatomy in rats illustrates extracranial and intracranial vascular relations exploited in our method of reversible occlusion of middle (Mid.) cerebral artery (a.). Vessel size is disproportionately enlarged for clarity. Cervical dissection is illustrated on the left. Common (Com.), external (Ext.), and internal (Int.) carotid arteries and their branches are shown.* Right: *Photograph of rat brain at autopsy showing intravascular suture, introduced through cervical internal carotid artery, within lumen of right anterior cerebral artery. Suture occludes origin of middle cerebral artery from intracranial internal carotid artery.*

suture has blocked the origin of the MCA, occluding all sources of blood flow from the ICA, ACA, and posterior cerebral artery (PCA). The incision was closed, leaving 1 cm of the nylon suture protruding so it could be withdrawn to allow reperfusion. Restoration of MCA blood flow did not require anesthesia. The suture was pulled back until resistance was felt, indicating that the tip had cleared the ACA–ICA lumen and was in the ECA stump, and then trimmed.

Bilateral vertebral artery occlusion (BVO) was performed using a modification of the technique described by Pulsinelli and Brierley.[16] Under a surgical microscope, the alar foramen was located at the lateral edge of C1 and enlarged with a dental drill. Each vertebral artery and its occipital branch were coagulated with microbipolar cautery forceps and divided.

In a subsequent series of experiments to confirm the optimum surgical technique, 34 younger rats weighing 300–400 g were anesthetized by inhalation of 1% halothane and subjected to one of five procedures (Groups A–E, Table 1). The occlusion technique was standardized by advancing a 4-0 suture, which had a rounded, slightly larger tip, exactly 17 mm into the ICA from the origin of the ECA in each rat.

Neurologic examinations were performed every 12 hours in Groups 1–8. In Groups A–E, neurologic examinations were performed 2, 4, and 8 hours after the onset of occlusion and then daily until sacrifice. The neurologic findings were scored on a five-point scale: a score of 0 indicated no neurologic deficit, a score of 1 (failure to extend left forepaw fully) a mild focal neurologic deficit, a score of 2 (circling to the left) a moderate focal neurologic deficit, and a score of 3 (falling to the left) a severe focal deficit; rats with a score of 4 did not walk spontaneously and had a depressed level of consciousness.

All surviving rats were killed 72 hours after the onset of occlusion, and the brains were removed and inspected to determine the position of the ICA suture. In 14 rats selected arbitrarily from Groups 1, 2, and 3, an India ink solution was injected into the ascending aorta before sacrifice to verify obstruction of anterograde blood flow to the territory of the MCA; the same technique was used to verify reperfusion of the MCA after reversal of the occlusion in six rats from Group 4. The absence or presence of staining of the MCA and its branches was considered proof of

Downloaded from http://stroke.ahajournals.org/ by guest on October 25, 2011

TABLE 2. Neurologic Deficit Score and Infarct Size After Intraluminal Suture Occlusion of Middle Cerebral Artery in 15 Rats That Survived 72 Hours

	Group B					Group C					Group D				
Neurologic score															
2 hr	2	1	2	2	1	2	2	2	2	2	2	1	2	1	2
4 hr	2	1	2	2	2	1	2	1	1	2	2	0	2	1	2
8 hr	0	1	2	1	2	1	2	1	1	2	1	0	1	1	1
24 hr	1	1	2	1	1	1	2	0	2	1	2	0	1	1	1
48 hr	1	1	2	1	1	1	2	0	1	1	1	0	1	1	1
72 hr	1	1	1	1	1	1	2	0	1	1	1	0	1	1	1
Area of ischemic injury on single coronal section at optic chiasm (%)															
Each rat	38.3	32.6	34.3	36.2	46.6	31.6	34.0	7.0	5.1	32.0	32.1	8.1	14.6	36.7	37.0
Group mean±SD	37.6±5.5					21.9±14.5					25.7±13.4				

Five rats from each group. Group B, permanent right middle cerebral artery occlusion; Group C, 2-hour right middle cerebral artery occlusion; Group D, 4-hour right middlé cerebral artery occlusion. Two rats in Group B were excluded from pathologic analysis because of inadequate brain fixation.

MCA occlusion or reperfusion. Some of the brains from the rats in the preliminary studies were sectioned coronally, incubated for 60 minutes in a 2% solution of 2,3,5-triphenyltetrazolium chloride (TTC) at 37° C for vital staining,[17–19] photographed, and fixed by immersion in 10% formalin solution. The remaining brains were placed in 10% formalin solution for later sectioning.

In Groups A–E, the brains were fixed by intracardiac perfusion of heparinized 0.9% saline followed by 10% formalin in a 0.1 M phosphate buffer (pH 7.4), removed, and stored in fixative. Blocks containing tissue from the anterior to the posterior edges of the corpus callosum were embedded in paraffin, cut into 6-μm sections, and stained with hematoxylin and eosin. Sections of interest were selected at 1-mm intervals and evaluated microscopically for ischemic tissue damage. Areas of neuronal injury or infarction were plotted on tracings from projections of the coronal sections. The area of neuronal injury or tissue necrosis was divided by the total area of the whole-brain coronal section as assessed by polar planimetry to obtain the percent infarcted area in each section. Average infarcted areas for each rat were calculated using three sections from each brain. This analysis showed no significant difference in mean infarct areas between Groups B, C, and D. Infarcted areas taken from a single section of each brain at the level of the rostral edge of the optic chiasm were then used to calculate the mean and standard deviations for each group. Nonparametric analysis (Kruskal-Wallis test) was used to determine significant differences in infarct size between groups.

Results

Cardiorespiratory function remained stable in all rats in the preliminary experiments. There was no significant alteration in Po_2 (90–120 torr), Pco_2 (28–45 torr), arterial pH (7.30–7.35), or systolic blood pressure (90–113 torr) during anesthesia; the average values were similar in all groups. The hemoglobin concentrations were 11–15 g% and varied little within or between groups.

EEG monitoring in the six rats in Group 5 showed a consistent bilateral decrease in amplitude after 2-hour temporary right MCA occlusion. After occlusion, the mean±SD EEG amplitude had declined to 25.4±28.5% and 69.2±16.0% of the baseline values in the ischemic and nonischemic hemispheres, respectively. After 2 hours of reperfusion, the mean±SD

TABLE 3. Pathologic Findings in 36 Rats in Preliminary Studies of Reversible Middle Cerebral Artery Occlusion Without Craniectomy in Rats

			Surviving rats		Dying rats
Group	*n*	Procedure	Suture position or vessel patency	TTC stain results	Suture position
1	8	R MCO	7 in ACA, 1 in intracranial ICA	6 of 8 infarcted	—
2	8	R MCO+L ICO	8 in ACA	6 of 8 infarcted	—
3	12	B VO+R MCO	10 in ACA	10 of 10 infarcted	1 in ICA, 1 perforated ICA
4	8	4-hr temporary MCO after B VO	No thrombosis in ICA	1 of 7 infarcted	1 perforated ACA

Two rats died of hemorrhage and one of pulmonary insufficiency. R MCO, right middle cerebral artery occlusion; L ICO, left internal carotid artery occlusion; B VO, bilateral vertebral artery occlusion; TTC, 2,3,5-triphenyltetrazolium chloride; ACA, anterior cerebral artery; ICA, internal carotid artery.

Downloaded from http://stroke.ahajournals.org/ by guest on October 25, 2011

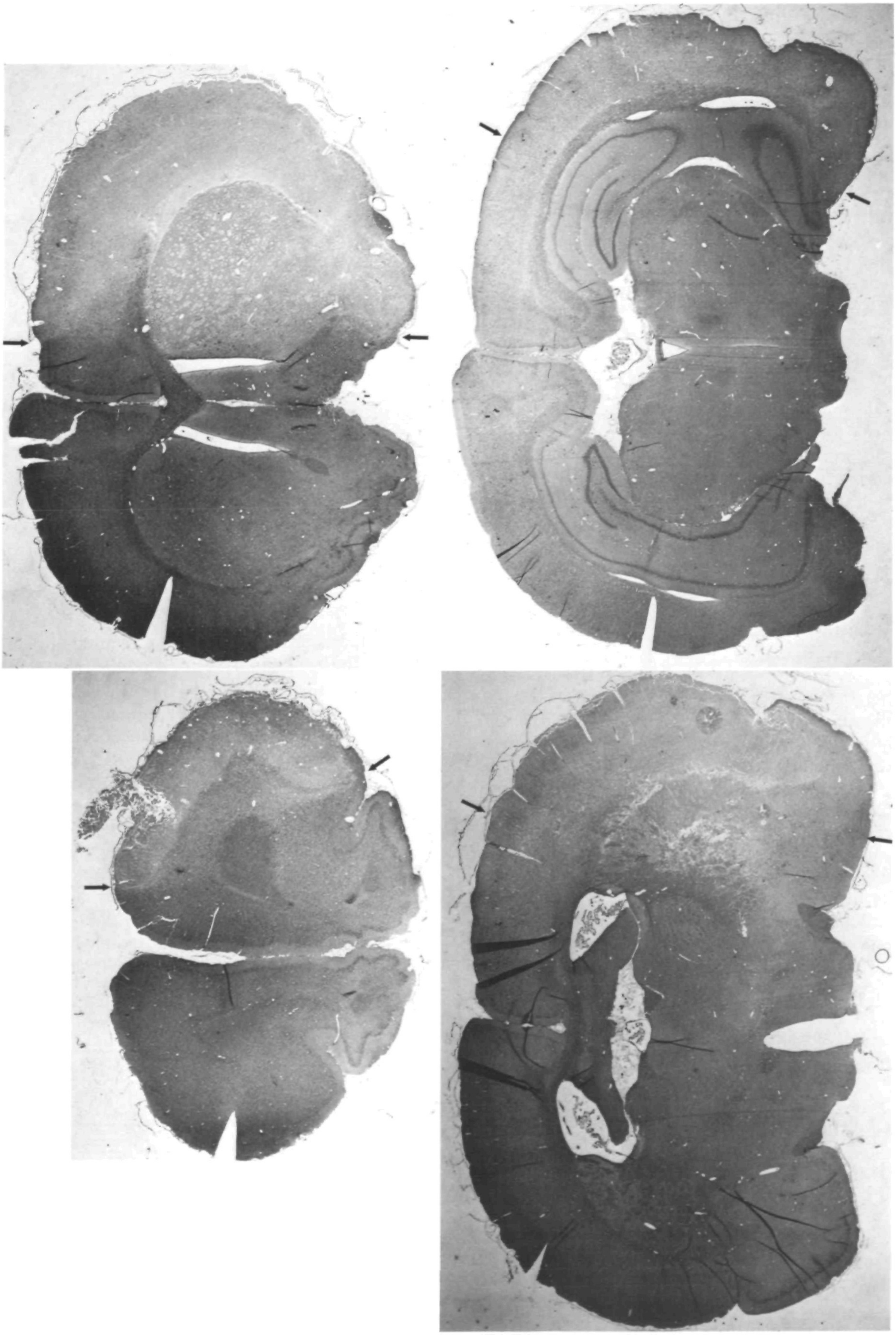

Downloaded from http://stroke.ahajournals.org/ by guest on October 25, 2011

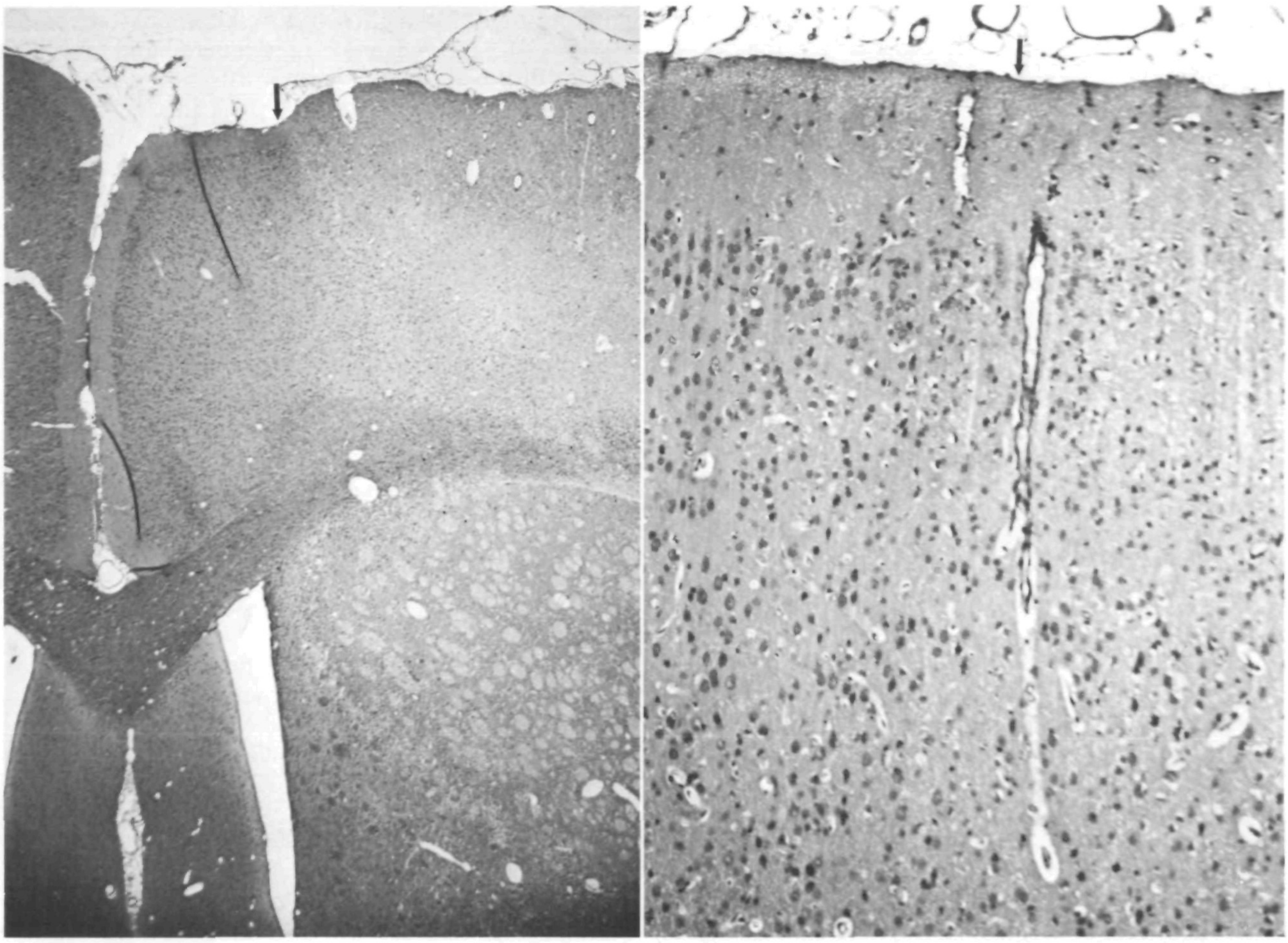

FIGURE 3. Left: *Low-power photomicrograph (×3.7) of infarct border zone in parasagittal region (arrow) shown in Figure 2, top right.* Right: *High-power photomicrograph (×18.5) of parasagittal infarct border (arrow) shown in Figure 2, bottom left. Note edema, nuclear pleomorphism, and pyknosis.*

EEG amplitudes were 36.4±25.9% of baseline in the ischemic hemisphere and 58.8±16.3% in the nonischemic hemisphere. Thus, EEG amplitude did not recover significantly after reperfusion. In one rat, EEG amplitude decreased in the ischemic hemisphere but was unchanged in the nonischemic hemisphere throughout occlusion and reperfusion.

Rats in Groups 1 and 2 had mild neurologic deficits at 12 hours after the onset of occlusion that resolved completely by 72 hours. In Group 3, moderate focal deficits (average score 2.3) were observed after full recovery from anesthesia (12 hours after the onset of occlusion); these deficits persisted at 24 hours, but score decreased to an average of 1.3 at 36 hours and remained stable until sacrifice at 72 hours after the onset of occlusion. The rats in Group 4 had no residual deficits.

The neurologic deficit scores for 15 rats in Groups B, C, and D are presented in Table 2. The average score 2 hours after the onset of permanent MCA occlusion (Group B) was 1.6, which decreased to 0.7 at 72 hours. There were no significant differences in neurologic deficit scores in Groups B, C, and D. No deficits were observed in Group A.

The procedures were fatal in five of 60 rats (8.3%) in the preliminary studies. Two of the 36 rats that underwent MCA occlusion in Groups 1–4 died of intracranial hemorrhage after the intraluminal suture perforated the ICA. The three other deaths (one in Groups 1–4) were from pulmonary insufficiency caused by an anesthetic overdose or airway obstruction during or after surgery.

In the confirmatory studies, there were no deaths in Group A. One of eight rats in Group B, one of six rats in Group C, and five of 10 rats in Group D died, all of neurologic deterioration. In Group E, three of five rats died before recovering from anesthesia.

FIGURE 2. *Photomicrographs of coronal sections of rat brain removed 72 hours after onset of 4-hour temporary occlusion of middle cerebral artery with intraluminal suture. Neurologic deficit score of 2 was observed after 2 hours of reperfusion; at sacrifice, deficit had improved to score of 1. Infarct area in right hemisphere (arrows) measured 37% of coronal brain section area averaged from sections taken 3 mm anterior to center of optic chiasm* (top left), *at level of optic chiasm* (top right), *and 1.5 mm* (bottom left) *and 4 mm* (bottom right) *posterior to optic chiasm.*

Downloaded from http://stroke.ahajournals.org/ by guest on October 25, 2011

In all but one rat in Groups 1–4, the suture had passed the ICA bifurcation and entered the ACA (Figure 1, right; Table 3). In 14 rats from Groups 1–3, obstruction of blood flow in the right ICA-MCA territory was verified by the absence of India ink staining. In the rats from Group 2, vessels in the territory of the left MCA were stained, presumably by collateral blood flow through the posterior circulation, even though no filling of the left ICA was observed. In six rats from Group 4, restoration of blood flow after withdrawal of the suture after 4 hours of ischemia was verified by India ink staining of the ICA and the MCA on the right side.

The results of vessel inspection and TTC staining in the 33 rats in Groups 1–4 that survived until sacrifice are also presented in Table 3. Sections through the ICAs and the ACAs at the level of the optic chiasm showed no evidence of thrombus or vessel wall damage in Group 4 after temporary MCA occlusion. No evidence of infarction was found in Groups 6, 7, or 8. The infarcts were well demarcated in Groups 1, 2, and 3, although the location was predominantly subcortical and the area was smaller in Groups 1 and 2. In Group 3, six of 10 rats had infarcts in the frontoparietal cortex and the subcortical basal ganglia in the distribution of the right MCA.

The confirmatory studies showed that the neuropathologic changes 72 hours after the onset of occlusion were more extensive in rats that underwent permanent occlusion than in those that underwent temporary occlusion (Table 2). Permanent MCA occlusion produced neuronal injury in 37.6±5.5% of the coronal section area, or nearly 75% of the hemispheric area (Figure 2). In Groups C and D, the mean±SD infarct sizes were 21.9±14.5% and 25.7±13.4%, respectively ($p<0.05$). Infarction in all three groups involved both cortical and subcortical regions (Figures 2 and 3). No neuronal injury was seen in Group A. Histologic sections suitable for analysis showed an infarct involving almost the entire right hemisphere in one of the two surviving rats in Group E.

Discussion

Cerebral ischemia restricted to the distribution of the MCA in rats has been achieved previously only by intracranial exposure and transection of that vessel.[12] With our method, a relatively simple extracranial microsurgical dissection of the ECA and the ICA and their branches near the base of the skull is performed to interrupt extracranial sources of collateral blood flow. Then, because rats do not have a rete mirabile, the lumen of the ICA and major potential sources of retrograde intracranial collateral blood flow can be blocked by cannulation with a blunted nylon suture introduced extracranially; MCA blood flow can be restored by withdrawing the suture. The neurologic deficit and the location and extent of infarction may have been less severe and less consistent in preliminary studies because older, larger rats with larger collateral blood vessels were used and because the diameter and length of the sutures inserted varied. In the confirmatory studies, the average infarct sizes in Groups B, C, and D (21.9–37.6% of coronal section area) verify that occlusion of the MCA by insertion of a 4-0 suture 17 mm beyond the CCA bifurcation reliably produces regional infarcts in 300–400-g rats under halothane anesthesia.

BVO followed immediately by occlusion of the right MCA (Group E) produced the most extensive ischemic insult but resulted in substantial blood and weight loss and a mortality rate of 60%. The addition of BVO presumably increased the severity of the ischemic injury by further reducing collateral blood flow from the posterior communicating artery and from transcortical connections between distal branches of the PCA and the MCA. Because occlusion of the left ICA and right MCA did not result in higher neurologic deficit scores or more extensive infarcts than BVO and MCA occlusion in our preliminary studies, we assume that transcortical and parenchymal collateral blood flow from the ipsilateral posterior circulation is more efficient than that from the contralateral carotid circulation. When the origin of the MCA was blocked with a 4-0 suture (Groups B, C, and D), retrograde blood flow from the PCA and the ACA was obstructed, interrupting collateral blood flow from these vessels and producing infarcts in both the basal ganglia and the cortex. We assume that use of a larger diameter (4-0) suture with a larger rounded tip inserted 17 mm (rather than 13–16 mm) beyond the CCA bifurcation in the confirmatory studies (Group B) in smaller rats more effectively blocked blood flow into the MCA than was the case in the preliminary studies (Group 1), in which BVO was required to produce consistent neurologic deficit, and infarct size (Group 3) after MCA occlusion was smaller and more limited in distribution.

In general, the anatomic distribution of the ACA, PCA, and MCA in rats is analogous to that in humans.[20] Although in rats the PCA arises directly from the proximal intracranial portion of the ICA, the posterior communicating artery connects the terminal cerebellar branch of the basilar artery with the PCA. The blood supply of the rat thalamus and basal ganglia is also similar to that in humans: contributions arise from the MCA, PCA, and recurrent branches of the ACA that parallel the olfactory tract.[21] The intraluminal suture obstructs blood flow to the basal ganglia from all but the recurrent branches of the ACA, which remain open to collateral perfusion from the left ACA and the left extracranial branches of the pterygopalatine artery that perforate the skull base. Thus, predominantly the middle and posterior portions of the caudoputamen region, including the internal capsule and the anterior thalamus, are rendered ischemic.

Only two of 71 rats subjected to MCA occlusion with this technique died of intracranial hemorrhage. Both of these hemorrhages occurred during the

Downloaded from http://stroke.ahajournals.org/ by guest on October 25, 2011

preliminary experiments; no such complications occurred after the surgical techniques were standardized. The India ink and histologic studies confirmed the restoration of patency of the ICA and MCA, but the possible occurrence of distal embolization or microcirculatory thrombosis cannot be eliminated on the basis of our limited histologic studies.

The failure of reperfusion to reduce neurologic deficits and improve the EEG in Groups C and D and the 50% mortality rate in Group D suggest that irreversible ischemic damage that was aggravated by reperfusion had already occurred. However, since infarcts were smaller after temporary than after permanent MCA occlusion, reperfusion may have had some benefit. The stability of arterial blood pressure and blood gases in the preliminary studies indicates that our method of extracranial cerebrovascular occlusion reliably produced regional ischemia without inducing hypotension or hypoxia. The bilateral EEG changes may have been caused by diaschisis after MCA occlusion.

Our preparation more closely models cerebrovascular occlusive disease than the method described by Bannister and Chapman,[14] in which hemispheric blood flow in rats is reduced by bilateral carotid artery ligation and creation of a unilateral arteriovenous fistula from the distal carotid stump to the jugular vein. Although ischemia can be reversed by occluding the fistula and removing the opposite carotid clip, bilateral hemispheric ischemia does occur, and the mortality rate was 90% when ischemia was reversed after 4 hours. Our results were comparable to those obtained by Koizumi et al[15] with a similar intraluminal suture technique.

Our model should prove useful for studying both permanent and temporary regional experimental cerebral ischemia to simulate cerebrovascular occlusive disease in humans[22] and for evaluating the effects of reperfusion and other physiological manipulations or treatments.

Acknowledgment

The authors thank Stephen Ordway for editorial assistance.

References

1. Brown AW, Brierley JB: Anoxic ischemic cell changes in rat brain: Light microscopic and fine structural observations. *J Neurol Sci* 1972;16:59–84
2. Dienel GA, Pulsinelli WA, Duffy TE: Regional protein synthesis in rat brain following acute hemispheric ischemia. *J Neurochem* 1980;35:1216–1226
3. Eklof B, Siesjo B: The effect of bilateral carotid ligation upon the blood flow and energy state of rat brain. *Acta Physiol Scand* 1972;86:155–165
4. Furlow TW, Martin RM, Harrison LE: Simultaneous measurement of local glucose utilization and blood flow in the rat brain: An autoradiographic method using two tracers labeled with carbon-14. *J Cereb Blood Flow Metab* 1983;3:62–66
5. Laas R, Igloffstein J, Meyerhoff S: Cerebral infarction due to carotid occlusion and carbon monoxide exposure. *J Neurol Neurosurg Psychiatry* 1983;46:756–773
6. Levine S: Anoxic-ischemic encephalopathy in rats. *Am J Pathol* 1960;36:1–17
7. Lear LJ, Ackermann R, Kameyama M: Multiple-radionuclide autoradiography in evaluation of cerebral function. *J Cereb Blood Flow Metab* 1984;4:264–269
8. Salford LG, Plum F, Brierley JB: Graded hypoxia-oligemia in rat brain. *Arch Neurol* 1973;29:227–238
9. Yanagihara T: Experimental stroke in gerbils: Correlation of clinical pathological and electroencephalographic findings and protein synthesis. *Stroke* 1978;9:155–159
10. Yoshimine T, Yanagihara T: Regional cerebral ischemia by occlusion of the posterior communicating artery and the middle cerebral artery in gerbils. *J Neurosurg* 1983; 58:362–367
11. Coyle P: Middle cerebral artery occlusion in the young rat. *Stroke* 1982;13:855–859
12. Tamura A, Graham ID, McCulloch J, Teasdale MG: Focal cerebral ischaemia in the rat. *J Cereb Blood Flow Metab* 1981;1:53–69
13. Tyson WG, Teasdale MG, Graham ID, McCulloch J: Cerebrovascular permeability following MCA occlusion in the rat. *J Neurosurg* 1982;57:186–196
14. Bannister CM, Chapman SA: Ischemia and revascularization of the middle cerebral territory of the rat brain by manipulation of the blood vessels in the neck. *Surg Neurol* 1984;21:351–357
15. Koizumi J, Nakazawa T, Yoshida Y: Reperfusable brain infarction model in the rat (abstract). *Proceedings of the 10th Meeting of the Japanese Stroke Society*, Kyoto, Japan, April 18–19, 1985, p 159
16. Pulsinelli WA, Brierley JB: A new model of bilateral hemispheric ischemia in the anesthetized rat. *Stroke* 1979; 10:267–272
17. Fallon JT: Simplified method for histochemical demonstration of experimental myocardial infarct. *Circulation* 1979; 60(suppl 2):11–42
18. Lie JT, Parolero PC, Holley KE, Titus JL: Macroscopic enzyme-mapping verification of large, homogeneous experimental myocardial infarctions of predictable size and location in dogs. *J Thorac Cardiovasc Surg* 1975;69:599–605
19. Bederson JB, Pitts LH, Nishimura MC, Davis RL, Bartkowski HM: Evaluation of 2,3,5-triphenyltetrazolium chloride as a stain for detection and quantification of experimental cerebral infarction in rats. *Stroke* 1986;17:1304–1308
20. Coyle P: Arterial patterns of the rat rhinencephalon and related structures. *Exp Neurol* 1975;49:671–690
21. Rieke KG, Bowers ED, Penn P: Vascular supply pattern to rat caudoputamen and globus pallidus: Scanning electronmicroscopic study of vascular endocast of stroke-prone vessels. *Stroke* 1981;12:840–846
22. Adams PM, Damasio CM, Putman FS, Damasio RA: Middle cerebral artery occlusion as a cause of isolated subcortical infarction. *Stroke* 1983;14:948–952

KEY WORDS • animal models • cerebral arteries • cerebral ischemia • rats

Downloaded from http://stroke.ahajournals.org/ by guest on October 25, 2011
